# A Technology System to Help People With Multiple Disabilities Increase Contact With Objects and Control Environmental Stimulation: Single-Case Research Design

**DOI:** 10.2196/70378

**Published:** 2025-03-21

**Authors:** Giulio E Lancioni, Gloria Alberti, Chiara Filippini, Nirbhay N Singh, Mark F O'Reilly, Jeff Sigafoos, Valeria Chiariello, Oriana Troccoli

**Affiliations:** 1 Lega F D'Oro Research Center Osimo Italy; 2 College of Medicine Augusta University Augusta, GA United States; 3 Department of Special Education University of Texas at Austin Austin, TX United States; 4 School of Education Victoria University of Wellington Wellington New Zealand

**Keywords:** technology, sensor, webcam, blindness, intellectual disability, motor impairment, object contact, stimulation

## Abstract

**Background:**

People with severe-to-profound intellectual disability and sensory-motor impairment tend to be passive and detached from their immediate context.

**Objective:**

This study assessed a new technology system using a webcam to detect participants’ responses (ie, hand contact with objects) and to trigger computer delivery of preferred environmental stimulation, such as music, contingent on (immediately after) the occurrence of those responses.

**Methods:**

In total, 8 adults with severe to profound intellectual disability and extensive motor and visual impairments participated in the study. Each participant was exposed to an ABACB design. The technology system did not provide stimulation during the A (baseline) phases, provided stimulation contingent on the responses during the B (intervention) phases, and provided stimulation throughout the sessions during the C (control) phase. Sessions lasted 5 minutes.

**Results:**

During the first baseline phase, the participants’ mean frequency of responses per session was between about 3 and 6.5. During the first intervention phase, it increased to between about 10 and 18. It showed a clear decline during the second baseline phase, remained low during the control phase, and increased again during the second intervention phase. During this phase, it ranged from about 13 to 19.5.

**Conclusions:**

The new technology system might be a useful tool to help people with intellectual and sensory-motor disabilities increase object contact and stimulation control.

## Introduction

### Background

People with severe-to-profound intellectual disability and sensory-motor impairment tend to be largely passive and detached from their immediate context [1-6]. Indeed, they may not have particular interest in the objects available to them, may lack speech and possess only minimal and inefficient forms of nonverbal communication, and may be unable to access environmental stimulation independently with the consequent risk of low and poor stimulation input [1-7].

This situation is largely unsatisfactory, and efforts have been reported to reduce its negative implications. Some of the efforts have been directed at increasing the level of interaction between these people and their staff and caregivers to increase their stimulation input and the opportunity of practicing basic forms of communication [2,6-12]. Other efforts have been more specifically focused on enriching these people’s daily conditions with increased levels of environmental stimulation [13-19]. These latter efforts presented a clear differentiation based on whether their scope was to (1) provide people with a rich stimulation context [18,20-22] or (2) enable people to exercise self-determination, thus seeking and controlling environmental stimulation through their own responses rather than relying on staff mediation [16,23,24]. Promoting self-determination can be considered a critically important goal within the rehabilitation process, that is, a goal that is instrumental to counter passivity (isolation) and foster independent contact with the immediate context and objects [25,26].

Studies aimed at enabling people to seek and control environmental stimulation independently (through their self-determination) have often relied on technology systems, including sensors (microswitches) linked to a computer, smartphone, or tablet [16,27-34]. Sensor activations via specific responses triggered the computer, smartphone, or tablet to deliver brief periods of preferred stimulation. In essence, the technology was arranged to ensure that even people with a limited response repertoire would always have 1 or 2 responses that they could use as tools (instruments) to seek and control environmental stimulation.

The results of those studies were largely encouraging. First, people were typically successful in using the responses selected for them to perform and thus enriched their stimulation input based on their own initiative (self-determination) and independent choice. Second, people seemed to enjoy such a situation. In fact, studies reported that they tended to show indices of happiness during sessions in which they were able to control their stimulation input through their responses [16,33,35-38]. Third, comparative evaluations seemed to indicate that indices of happiness tended to be higher (more frequent) in situations in which people controlled the stimulation through their responses than in situations in which the stimulation was automatically delivered (ie, independent of people’s responses) [16,36].

The results mentioned earlier constitute an important basis for supporting the use of technology-based interventions aimed at enabling people to independently seek and control their environmental stimulation. Extending the use of those interventions poses questions about the responses the participants should be required to produce and the sensors that could be used to detect those responses. As to the responses, it would seem advantageous to select those that involve people’s physical contact with objects (ie, to curb their isolation and detachment). As to the sensors, the main question is to ensure that they are suitable and dependable in detecting responses involving touching or exploring objects. The answer to this question is not always obvious. For example, one may argue that optic sensors placed in the proximity of the objects to be touched or explored could easily detect the responses. Yet, response detection might become inaccurate if the sensors’ focus is altered during the sessions by people’s erratic response movements. Pressure sensors under or to the side of the objects to be touched and explored could be effective. Yet, that would require that people apply some pressure on the objects.

### Objectives

The purpose of this study was to assess (1) the suitability (applicability) of a sensor that did not need to be displayed in the proximity of the objects to be touched or explored but would detect the responses from a distance, thus bypassing the difficulties mentioned regarding optic and pressure sensors, and (2) the effectiveness of the sensor and related technology system in helping the participants increase contact with objects and control environmental stimulation. A positive answer regarding each of these 2 assessment points was thought to have relevant practical (clinical) implications for future work with people with multiple disabilities. The sensor consisted of a webcam. The technology system of which the sensor was part involved a portable computer fitted with specific software, a mini speaker, and a smart Wi-Fi plug. In total, 8 people with severe to profound intellectual disability and sensory-motor impairments were involved in the study, which was carried out using single-case research methodology.

## Methods

### Participants

The 8 participants represented a convenience sample [39] in that they were selected from rehabilitation and care centers of a single organization. All of them, however, shared a complex condition in terms of disabilities and limited engagement with the immediate context and were adults. Table 1 lists the 8 participants via their pseudonyms and reports their chronological age and their Vineland age equivalents for Daily Living Skills, personal subdomain (only this subdomain of the Vineland Adaptive Behavior Scales was used as it seemed the most representative of their general functioning). Their chronological age ranged from 27 to 48 years. Their Vineland age equivalents (obtained via the second edition of the Vineland Scales [40,41]) ranged from below 1 year to 2 years and 2 months, underlining the seriousness of their situation and their dependence on external support. All participants had congenital encephalopathy and presented with intellectual disability, motor impairments (ie, lack of ambulation or ambulation with support and arm-movement restrictions), absence of speech or any formal communication means, and minimal residual vision (Logan and Harper) or blindness (all others). No IQ scores were available for them as no formal testing was possible given their situation. The psychological services of the rehabilitation and care centers that they attended estimated their level of intellectual disability to fall in the profound or severe-to-profound range.

**Table 1:** Participants’ chronological age and Vineland age equivalents for Daily Living Skills (personal subdomain).

Participants (pseudonyms)	Chronological age (years)	Vineland age equivalents^a^ (years, months)
Liam	43	2, 2
Hallie	37	<1, 0
Logan	31	<1, 0
Kali	48	1, 3
Harper	43	2, 1
Jacob	33	1, 5
Millie	27	1, 1
Isabel	34	1, 2

^a^Age equivalents are based on the Italian standardization of the Vineland Scales [40].

The participants were included in the study based on a number of conditions, which had been verified through preliminary observations and staff interviews. First, they were generally passive with very limited contact with their immediate context but possessed the arm-hand motor schemes necessary to reach and touch objects on their desk. Second, they showed signs of interest in forms of environmental stimulation (eg, could display alerting and smiling in relation to music and songs). The assumption was that such stimulation could be used contingent on their object contact responses during the study. Third, they seemed to alert and sometimes to activate themselves (eg, producing a reaching response) in relation to the presentation of alerting stimuli such as verbal encouragements or noises. Fourth, rehabilitation personnel considered an increase in the participants’ responses highly useful to break their withdrawal and to promote functional motor schemes. Fifth, the use of a technology system to help the participants acquire and consolidate their responses was viewed favorably within their daily contexts by regular staff and caregivers.

### Setting, Sessions, Responses, Research Assistants, and Stimuli

Quiet rooms of the rehabilitation and care centers that the participants attended were used as the setting for the study sessions. In total, 3 types of sessions were available, that is, baseline, intervention, and control sessions. All sessions lasted 5 minutes. They were implemented on an individual basis, typically 2-3 times a day (nonconsecutively), 3-6 days a week. A response consisted of the participants making a new hand contact with (ie, touching or exploring) either one of the 2 objects available in front of them, that is, on the desk at which they sat. The objects included simple everyday materials such as sponges, small boxes, rings, and bottles, which were fixed on the desktop.

In total, 4 research assistants were responsible for implementing the sessions and checking their agreements and disagreements with the technology system regarding the responses it recorded and followed with stimulation delivery. They had university degrees in psychology and were familiar with the implementation of technology-aided programs with people with disabilities and with data recording procedures. Expert research assistants were involved in carrying out the study because they were expected to need only minimal practice (preparation) time and to be procedurally reliable given their experience. Moreover, contrary to staff personnel, they did not have care and rehabilitation duties that could interfere with the timing and implementation of the sessions.

The stimuli used during the intervention and control sessions included music, songs, noises, and voices or combinations of them with various types of lights (Logan) or mild airflows (Millie). The stimuli had been selected through a stimulus preference screening procedure carried out before the start of the study. The procedure consisted of presenting each of three 10-second segments of the songs and music pieces as well as clips of different lights, voices, and noises or brief airflows for at least 10 nonconsecutive times (ie, over different assessment periods). The stimuli were retained for the intervention and control sessions only when the research assistants and staff members involved in the screening agreed that at least 50% of their presentations were followed by positive reactions (eg, orienting and smiling) [26].

### Technology System

The technology system was similar to that used by Lancioni et al [28] and included a webcam sensor linked to a portable computer, a Bluetooth mini speaker, and a smart Wi-Fi plug. The smart Wi-Fi plug was used only when the preferred stimuli following the responses included lights or airflows (see the *Setting, Sessions, Responses, Research Assistants, and Stimuli* section). The computer was fitted with Windows 11 and specific software. The webcam was mounted on a tripod to monitor the participants’ responses from a distance. At the start of the study, the research assistants determined the best position of the webcam for each participant so that it could provide the computer and related software with a clear image of the participant’s face and hands and of the objects to be touched. The software, which is freely available [42], was developed using the Python programming language and built on open-source libraries. These included OpenCV for image processing, MediaPipe Pose for detecting human body landmarks (specifically, hand landmarks) in 3D space, and python-kasa for controlling the smart Wi-Fi plug.

The software enabled the system to perform a number of essential functions. First, the system (1) monitored (via the webcam) the position of the participants’ hands relative to the objects placed in fixed positions on the desk during all study sessions, and thus (2) could determine whether the participants performed object contact responses by comparing the hand landmarks with the object positions. Second, the system provided participants with a 10-second period of preferred stimulation, such as music, contingent on (immediately after) each response performed during the intervention sessions. During the 10-second stimulation periods (and, for consistency, the 10-second period following each response in all other study sessions), the system halted its hand position monitoring so that no new response was recorded during that time. Third, the system controlled the presentation of alerting events during the different phases of the study (see the *Baseline I* section). Fourth, the system memorized the session setup parameters recorded for each participant at the start of the study (following the positioning of the webcam) so that the same parameters could be applied across all sessions. Fifth, the system assisted in recording the frequency of responses that occurred in the sessions. Preferred stimulation and alerting events were presented via the Bluetooth mini speaker, which served to increase their volume.

### Measures and Data Recording

The first measure concerned the frequency of responses the participants performed during the baseline, intervention, and control sessions and was recorded via the technology system. The second measure concerned the level of research assistants’ agreement with the system on the responses the system recorded and followed with stimulation during about 50% of the sessions of Intervention II. In connection with each of those responses, the research assistants were to note whether they did or did not agree with the system’s recording. The percentage of agreement between research assistants and the system (computed on single sessions by dividing the agreements by the agreements plus disagreements and multiplying by 100%) ranged between 88% and 100%, with means for the single participants exceeding 96%.

### Experimental Conditions and Data Analysis

#### Overview

Each participant was exposed to an ABACB design, in which A represented the baseline condition, B represented the intervention condition (with the system delivering stimulation contingent on the participants’ responses), and C was a control condition [43]. The first baseline (A) phase included different numbers of sessions for the different participants according to a nonconcurrent, multiple baseline design across participants [43]. This single-case research design format was considered adequate to determine the strength and internal validity of the data gathered in the study [43].

To ensure a high level of accuracy from the research assistants (ie, a high level of procedural fidelity [44]) during the implementation of the 3 types of sessions, 2 strategies were adopted. The first strategy consisted of the research assistants practicing the use of the technology system prior to the start of the study. This was to enable them to determine the best position of the webcam and setup of the system with the participants. The second strategy consisted of the availability of regular feedback for the research assistants. Specifically, they were informed as to whether or not they were accurate in their implementation of the procedural conditions by a research supervisor who had access to video recordings of the sessions. This feedback was viewed as a precautionary measure more than a necessity given the research assistants’ initial practice with the system and the fact that the system memorized the participants’ session parameters and followed (enacted) those parameters automatically.

The participants’ frequency of responses across the study phases was summarized in graphic form. The differences in response frequency between the (1) first baseline and first intervention phase, (2) second baseline and second intervention phase, and (3) control phase and second intervention phase were assessed using the percentage of data points exceeding the median (PEM) method [45,46]. This method, which is a basic and practical tool for the evaluation of within-subject research data, served to determine for each participant the percentage of intervention sessions with a frequency of responses higher than the median of the previous baseline or control phase.

#### Baseline I

**Figure 1:**
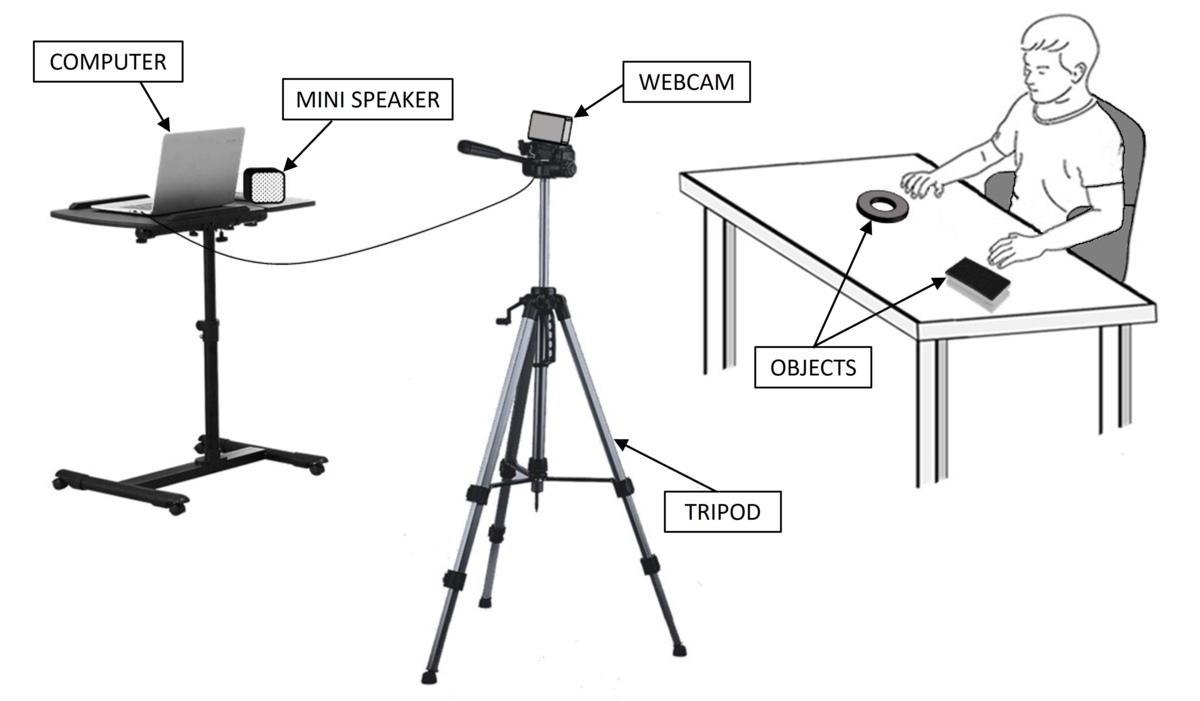
Schematic representation of a participant sitting at a desk with objects and of the webcam, computer, and mini speaker.

Baseline I included 5-10 sessions. The participants sat at a desk that contained 2 easily reachable objects; that is, they were in a situation familiar to them (Figure 1). Before the start of a session, the research assistants guided the participants (through physical prompts, which could be accompanied by a verbal expression such as “Touch here”) to touch the objects once or twice; that is, they provided 1 or 2 response practice trials. In an attempt to avoid the risk that the participants would remain passive throughout the sessions, alerting events were presented after periods of 30-40 seconds of no responding. These events (ie, 2-word verbal encouragements or brief sounds and noises triggered by the computer and delivered via the mini speaker) were to enhance the participants’ vigilance and attention and eventually facilitate some responding (see the *Participants* section). The technology system was available (see Figure 1 for a schematic view of the webcam, computer, and mini speaker) but only served to present the alerting events and record the responses. In fact, no stimulation was scheduled for the responses.

#### Intervention I

Intervention I included 15-31 sessions. Conditions were as in Baseline I with the difference that the system provided the participants with 10 seconds of preferred stimulation (eg, music and song clips) after each response. The first session was preceded by 5-7 response practice trials with stimulation following each response. Intervention I continued until the participants had shown a clear response increase.

#### Baseline II

Baseline II included 5-9 sessions. Conditions were as in Baseline I.

#### Control Phase

The control phase included 5-8 sessions. Conditions were as in Baseline II with regard to the availability of objects and alerting events. The difference was that the participants were provided with stimulation throughout the sessions. Stimulation changed several times during the session to minimize the risk of habituation effects [16]. The control phase was included to determine if stimulation availability per se was responsible for the participants’ increased responding, that is, if responding was the result of general activation (excitation).

#### Intervention II

Intervention II included 43-74 sessions. Conditions were as in Intervention I. During about half of the sessions, research assistants were to note whether they did or did not agree with the system regarding each of the responses the system recorded and followed with stimulation delivery (see the *Measures and Data Recording* section).

### Ethical Considerations

Staff and caregivers considered the study a positive opportunity for the participants. In fact, the study was intended to help them acquire and practice functional motor responses and access preferred stimulation within a comfortable session arrangement that was free from any specific risk. The participants’ legal representatives (who were contacted given the participants’ inability to give their consent to the study) fully agreed with the staff and caregivers’ view. They signed a consent form authorizing the participants to be involved in the study with (1) the possibility of ending such involvement at any time and (2) the guarantee of data deidentification. No participant compensation was available. The study complied with the 1964 Helsinki Declaration and its later amendments and was approved by the Ethics Committee of the Lega F. D’Oro, Osimo (AN), Italy (P030820241).

## Results

**Figure 2:**
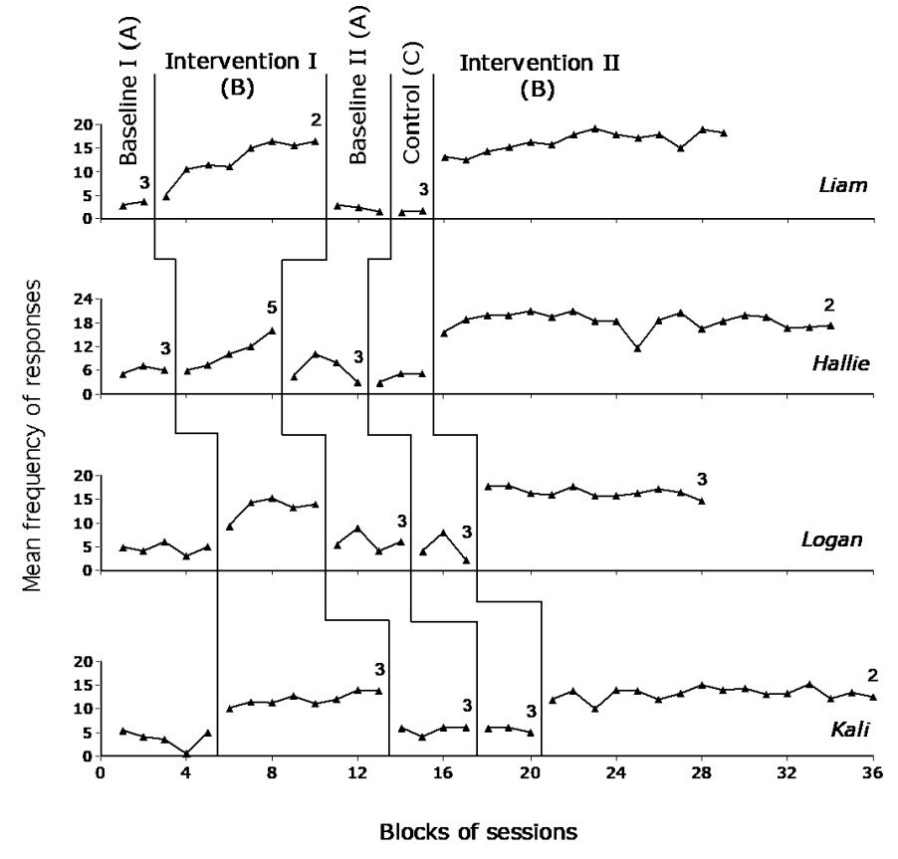
Data points for Liam, Hallie, Logan, and Kali. Each data point represents the mean frequency of responses per session over a block of sessions. The blocks include 2 sessions during the baseline and control phases and 4 sessions during the intervention phases. Blocks with numbers of sessions differing from those mentioned earlier are marked with a numeral, which indicates how many sessions those blocks include.

**Figure 3:**
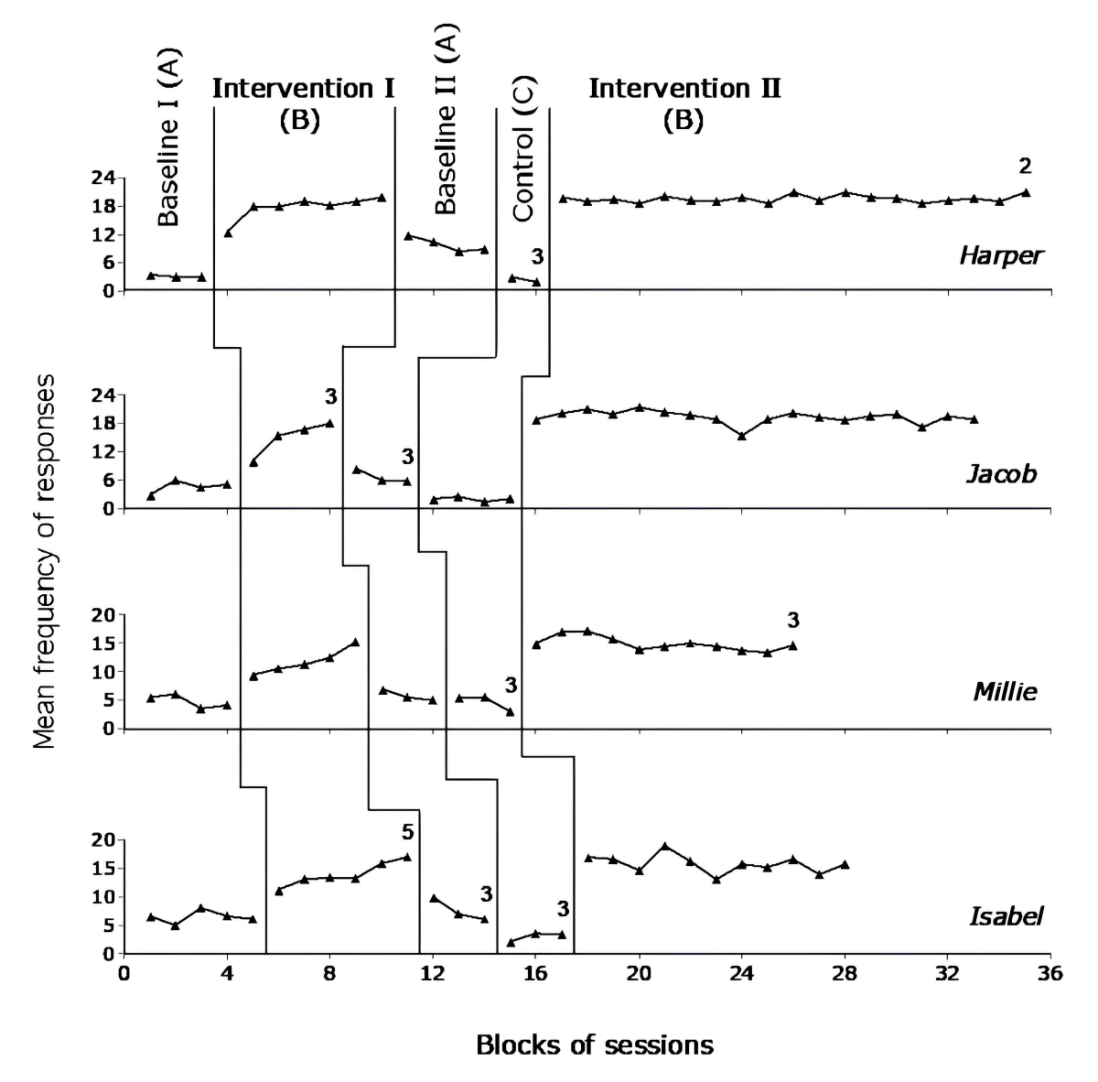
Data points for Harper, Jacob, Millie, and Isabel. Each data point represents the mean frequency of responses per session over a block of sessions. The blocks include 2 sessions during the baseline and control phases and 4 sessions during the intervention phases. Blocks with numbers of sessions differing from those mentioned earlier are marked with a numeral, which indicates how many sessions those blocks include.

Figures 2 reports the data for Liam, Hallie, Logan, and Kali and Figure 3 reports the data for Harper, Jacob, Millie, and Isabel over the different phases of the study. Each data point represents the mean frequency of responses per session over a block (group) of sessions. The blocks, which are used to simplify the graphic presentation of the data, include 2 sessions during the baseline and control phases and 4 sessions during the intervention phases. Blocks with numbers of sessions differing from those mentioned earlier are marked with a numeral, which indicates how many sessions those blocks include.

During Baseline I, the participants’ frequency of responses per session varied between 0 and 10, with means ranging from about 3 (Harper) to 6.5 (Isabel). During Intervention I, all participants showed a clear response increase. Their frequency of responses per session varied between 5 and 23, with means ranging from about 10 (Hallie) to 18 (Harper). The PEM method used to compare the data of Intervention I with those of Baseline I provided indices of 0.86 to 1. These indices pointed out that the response frequency of all or nearly all Intervention I sessions was above the median values of Baseline I, thus confirming the positive impact of the intervention conditions.

During Baseline II, the response frequency decreased for all participants. Their frequency per session varied between 1 and 14. Their means ranged from slightly above 2 (Liam) to about 10 (Harper). The response frequency did not seem to increase during the control phase. In fact, several participants tended to have a decline in responding. During Intervention II, the participants showed a response frequency similar to that observed toward the end of Intervention I. Their mean frequency ranged from about 13 (Kali) to 19.5 (Harper). The PEM method used to compare the data of Intervention II with the data of Baseline II and of the control phase provided indices of 0.98 to 1. These indices pointed out that the response frequency of all or nearly all Intervention II sessions was above the median values of Baseline II and of the control phase, thus confirming the strong impact of the intervention conditions. The difference between the intervention condition and the control phase indicates that the stimulation contingent on the participants’ responses (and not the availability of stimulation per se) was responsible for the response increase.

The research assistants’ level of agreement with the system on the responses that the system recorded and followed with stimulation delivery was high. Indeed, the percentages of agreement (already reported as part of the data recording process; see the *Measures and Data Recording* section) ranged between 88% and 100%, with means for the single participants exceeding 96%. It may also be added here that no specific system dysfunctions were reported during the study and that the research assistants were successful in using it. In fact, they quickly managed to (1) determine the best position of the webcam for each participant (ie, the position that provided the system with a clear image of the participant’s face and hands and of the objects to be touched), (2) save the related parameters in the system, and (3) apply those parameters at the start of all study sessions to ensure reliability across them.

## Discussion

### Principal Findings

The results indicate that the webcam-based sensor was adequate to ensure monitoring of the participants’ responses, and the technology system in its entirety was effective in helping the participants increase the frequency of those responses [16,27]. The dependability of the sensor was confirmed by the high percentages of agreement that research assistants had with the system regarding the responses it recorded and followed with stimulation delivery. The effectiveness of the system in its entirety was underlined by the differences in participants’ response performance between the intervention phases and the baseline and control phases. The fact that the study sessions were implemented by expert research assistants (see the *Setting, Sessions, Responses, Research Assistants, and Stimuli* section) does not imply that regular staff would not be as successful following a brief practice period with the technology.

Using a sensor that is largely unobtrusive (ie, that does not need to be connected to the participants’ body or to the objects that the participants are to touch and explore) can be considered an important practical advantage compared to using conventional sensors such as touch and optic sensors [47-50]. The latter sensors, in fact, although profitably used in the past [3,27,34], need to be arranged in contact with or proximity to the objects targeted for the participants’ responses, with the risk that those responses may alter their position and make their functioning inaccurate. Accessibility to the sensor and technology system reported may be facilitated by the fact that (1) the webcam, portable computer, Bluetooth mini speaker, and smart Wi-Fi plug are commercial devices, and (2) the software is freely available. The cost of the system in its entirety is about US $850. This cost might be viewed as relevant for a single user and quite reasonable for a rehabilitation center in which several participants could benefit from the system. One might also expect that cheaper and simpler versions of the system will be developed in the near future, given the great demand for technology support in this area [29,30,51].

The existence of a reliable sensor, applicable in situations in which other (conventional) sensors such as touch, pressure, and optic sensors may be difficult to use, can be considered an important step forward in the development of technology-aided programs for people with extensive multiple disabilities (ie, people who tend to be passive and largely dependent on staff and caregivers for accessing environmental stimulation). Indeed, a reliable and suitable sensor can enable care and rehabilitation staff to help participants strengthen a developmentally relevant behavior, such as contact with the immediate environment, in a substantially independent (self-determined) manner [52-55].

Pursuing participants’ independent contact with their immediate context and control of environmental stimulation may be viewed as a significant clinical and rehabilitation objective within any intervention program for people with extensive disabilities [27,56-58]. In fact, people who make contact with and explore objects in their immediate proximity (1) exercise useful motor responses that curb their tendency to be passive and detached, and (2) discover the power of their responses through the stimulation following those responses [54,59-61]. This discovery can then help them maintain their responses over time (strengthening their self-determination) and contribute to improving their appearance, mood, and quality of life [16,55,61-64].

While no direct data were collected regarding the participants’ mood during the intervention sessions of this study, evidence from other studies in the area suggests that intervention conditions may have a positive impact on mood [16,33,37,38,65,66]. As to the discovery of the power of one’s own responses, the data of this study add useful information. The participants’ consistent responding during the intervention phases and the low responding during the control phase indicate that responding was not simply the consequence of stimulation availability. Rather, it appeared to be linked to the participants’ discovery of the stimulation contingency value or, in other words, of their responses’ power to control stimulation occurrence [60,61,63]. Such a discovery could be taken as a clear sign of clinical and rehabilitation progress [58,62].

Intervention approaches based on the use of a technology system like that used in this study or a new version of it may have relevant implications also for staff personnel. In fact, the system would allow them to offer participants extra opportunities for positive engagement with relatively limited time investment. This could be viewed as an extension and enrichment of the intervention protocol with affordable extra costs in contexts where staff resources are typically limited.

### Limitations and Future Research

The study presents 2 main limitations, that is, the absence of maintenance and generalization data, and the lack of a social validation check. The first limitation prevents one from making statements as to whether the system can be effectively used over time and across different contexts. To amend this limitation, future studies will have to extend the data collection to longer periods of time using a variety of objects as well as different settings [60,61,67,68]. Positive maintenance and generalization data would provide a strong basis for considering the system a profitable tool within an intervention protocol designed for people with extensive disabilities. Support for the system might be further strengthened by the recognized need for technological assistance within programs directed at people with extensive and multiple disabilities [27,29,30,49,51].

The second limitation prevents one from making statements as to how the system may be viewed by staff, caregivers, and other service providers working within daily contexts for people with extensive disabilities. One way to address this limitation is to arrange surveys with care and rehabilitation personnel about the system’s effectiveness, acceptability, and applicability in daily contexts. Surveys could be carried out by having the personnel (1) view video clips of intervention sessions carried out with different participants, and (2) rate the content of those clips in terms of system effectiveness, acceptability, and applicability [66,69,70].

One might also find the relatively small number of participants and the use of short (5-min) sessions to be additional limitations of the study. As to the first of these 2 potential limitations, one may argue that the single-case experimental methodology used with the 8 participants to evaluate the impact of the system was adequate to confirm the internal validity of the data reported [42,71,72]. Single-case replication studies and studies using group designs could provide new important evidence to determine the external validity of the present findings [71-73]. Regarding the short sessions, 2 views may be expressed. On the one hand, it may be argued that the use of short sessions represents a limitation of the study that does not allow one to determine for how long people like our participants can remain positively engaged with objects. On the other hand, it can be stated that, given the typically limited attention of these people [74,75], using short sessions may be a largely justified choice.

### Conclusions

The results indicate that the technology system used in the study was adequate to ensure monitoring of the participants’ responses (ie, touching and exploring objects) and to control the automatic delivery of preferred environmental stimulation contingent on those responses. A sensor that is largely unobtrusive and does not need to be near or physically connected to the objects to be reached (1) can be considered advantageous compared to conventional sensors such as touch and optic sensors, and thus (2) can allow new intervention opportunities for people with extensive disabilities. While highly encouraging, these results are to be taken with caution until new research evidence is available, and the limitations of this study have been addressed.

## Abbreviations

PEM: percentage of data points exceeding the median

## Data Availability

The datasets generated during this study are available from the corresponding author on reasonable request. The software the authors developed to control the experimental conditions is available at no cost [41].
